# Early Profound Secondary Autoimmune Thrombocytopenia Induced by Clopidogrel in a Patient with a Coronary Artery Stent

**DOI:** 10.5505/tjh.2012.59244

**Published:** 2012-03-05

**Authors:** Volkan Karakuş, Burak Deveci, Erdal Kurtoğlu, Şakir Arslan

**Affiliations:** 1 Antalya Research and Training Hospital, Department of Hematology, Antalya, Turkey; 2 Antalya Research and Training Hospital, Department of Cardiology, Antalya, Turkey

## TO THE EDITOR

Clopidogrel in combination with aspirin is commonlyused for the prevention of thrombosis in patients with coronaryartery stents [1]. Moreover, since being introducedclopidogrel has proven to be very safe, tolerable, and efficacious[2]. Indications for clopidogrel use are expanding,and include reduction of such cardiovascular events asmyocardial infarction and stroke in appropriate patients.As the use of clopidogrel increases, uncommon side effectsare encountered more frequently [3]. Autoimmune thrombocytopeniais an extremely rare adverse effect of clopidogreltherapy [4]. We report a case of autoimmune thrombocytopeniarelated to standard clopidogrel treatment in apatient 2 days after coronary stenting.

A 63-year-old male presented with acute inferior myocardialinfarction, which was treated with primary stenting;heparin was administered periprocedurally. Thepatient was given aspirin (300 mg d^–1^ p.o.) and clopidogrel(75 mg d^–1^ p.o.), and 2 days later he developed diffusepetechiae that prompted evaluation. Physical examinationdid not reveal any signs of splenomegaly. Laboratory findingswere as follows: platelet count: 7 x 10^9^ L^–1^; hemoglobin:13.4 g dL–1; leukocyte count: 4.7 x 10^9^ L^–1^. Peripheralblood smear showed severe thrombocytopenia withoutmicroangiopathic changes. The lactate dehydrogenaselevel, prothrombin time, and activated partial thromboplastintime were normal. The diagnosis of clopidogrelassociatedautoimmune thrombocytopenic purpura wassuspected.

Clopidogrel and aspirin were discontinued at the timeof application. Intravenous methylprednisolone (1 g) wasgiven daily. A total of 2 units of platelets were transfusedin the first day of treatment. The patient responded in thethird day of high dose steroid therapy, with stabilizationof the platelet count (45 x 10^9^ L^–1^) and no new petechiae.According to the guidelines of The American Society ofHematology, bone marrow biopsy and aspiration were notperformed because bone marrow examination is not recommendedin cases of isolated thrombocytopenia highlysuspicious for the diagnoses of immune thrombocytopenicpurpura [5]. Aspirin was subsequently resumed in thethird day of high dose steroid therapy and high dose steroiddiscontinued then oral methylprednisolone 1mg/kgwas administered. 6 days later the platelet count was 132x 10^9^ L^–1^ while on oral steroid therapy Written informedconsent was obtained from the patient for publication.

Thrombocytopenia is a rare, but dangerous adverseeffect of clopidogrel, and encompasses thrombotic thrombocytopenicpurpura (TTP), isolated thrombocytopenia,and autoimmune thrombocytopenia [2]. Autoimmunethrombocytopenic purpura is an immune regulation disorderin which autoantibodies cause platelet destruction[6]. Treatment targets the prevention of splenic plateletdestruction, using such medications as corticosteroids,intravenous immunoglobulin, and azathioprine. Splenectomyis indicated when medical therapy fails [3]. The presentedpatient responded to high-dose steroid treatment.The patient’s response to steroid therapy confirmed anautoimmune etiology, which together with the time framesuggested it was unlikely to have been related to heparin.Careful monitoring for hematologic adverse effects followingclopidogrel use is essential for prompt diagnosis andtreatment of this potentially life-threatening complication.

## CONFLICT OF INTEREST STATEMENT

The authors of this paper have no conflicts of interest,including specific financial interests, relationships, and/or affiliations relevant to the subject matter or materialsincluded.
